# NOVEL DATA AND APPROACHES TO THE STUDY OF HEALTH AND AGING IN NSHAP

**DOI:** 10.1093/geroni/igz038.2968

**Published:** 2019-11-08

**Authors:** Linda J Waite

**Affiliations:** University of Chicago, Chicago, Illinois, United States

## Abstract

The National Social Life, Health, and Aging Project (NSHAP) is a longitudinal, population-based study that seeks to improve an understanding of the well-being of older, community-dwelling Americans. It accomplishes this by affording researchers a wide range of high quality measures that enable examining interactions among physical health and illness, medication use, cognitive function, emotional health, sensory function, health behaviors, social connectedness, sexuality, and relationship quality. The panelists in this symposium use NSHAP data to shed light on previously un- or underexplored aspects of health during aging. Kaufman et al. use interviewer ratings of respondents’ skin shade along with respondents’ individual experiences of discrimination, neighborhood racial composition, and other factors to characterize heterogeneity in the racial experience and how heterogeneity relates to health inequities. Riley integrates information on respondents’ residential region at birth and in older age to show that older adults who left the South are less healthy than those who stay in the South, and that social embeddedness helps to explain the health benefits for those who stay. Huang et al. take advantage of rich structural and functional social connectedness data to show that self-reported hearing impairment is associated with depth but not breadth of social connections. Huisingh-Scheetz et al. capitalizes on performance measures of gait speed and chair stands obtained at each wave to examine whether repeated measures improve the ability to predict loss of independence in activities of daily living. Discussant will discuss the importance, strengths, and weaknesses of these papers, and consider implications for future research.

